# Bringing X, Y, Z Generations Together to Facilitate School-Based Sexual and Reproductive Health Education

**DOI:** 10.5539/gjhs.v8n9p132

**Published:** 2015-10-29

**Authors:** Mahnaz Akbari Kamrani, Sharifah Syed Yahya

**Affiliations:** 1 Faculty of Nursing & Midwifery, Alborz University of Medical Sciences, Karaj, Iran; 2 Faculty of medicine and Health Sciences, University Putra Malaysia, Malaysia

**Keywords:** barriers, Malaysia, science teacher, sex education, school-based education, sexual and reproductive health, qualitative study

## Abstract

This generic qualitative study explores the perspective of Malaysian teachers regarding the constraints of the current school-based sexual and reproductive health education in secondary schools of Klang-Valley Malaysia. For this study, in-depth interviews were conducted with twenty eight science teachers of government schools. The majority of participants named the teaching strategy and capacity of teachers, the lack of co-operation from the school and parents, limited resources in teaching and students themselves as some of the challenges. We concluded that if sexual health education is to be effective, it needs to be provided by people who have some specialized training. The teachers should be trained to teach sexual reproductive health education classes at the basic level, and in-service training for teachers already in the field should be intensified. Local adaptation to culture, language, religion, and so forth is often necessary.

## 1. Introduction

Despite making up a large proportion of the population in developing countries, adolescents often have little choice relating to their sexual and reproductive health, lacking the information, skills, and services necessary to make informed decisions (Akbari-Kamrani, 2011). Research shows that education plays an important task in guiding and changing young people’s behavior (Akbari-Kamrani, 2011; Anwar et al., 2010, NDHS, 2006). Such school programs, if tactfully designed and well implemented, can provide young people with a solid foundation of knowledge and skills (Akbari-Kamrani, 2011; Rosen et al., 2004).

According to Rosen et al. (2004), school -based sexual and reproductive health education (SBSRHE) is one of the most important and effective ways to help young people manage their reproductive health. The rationale includes the opportunity offered by the schools’ curriculum to provide accurate, comprehensive and appropriate information on reproductive and sexual health that is associated with delayed premarital sex and lower rates of unwanted pregnancy and STIs in many countries (Pokharel et al., 2006). Second, the school provides notable opportunity for sex education as the major anatomical and physiological development of the child takes place during this period, and the child can be helped to learn about them and develop beneficial practices. Furthermore, the peer group situation exists in schools which could be used to effectively teach sexuality formally or informally. Finally, adolescents generally refer to the school as an information source on sexuality, valuing not only this knowledge, but also the place where it was received (Abraham et al., 2004). The challenge is to extend and fortify school based programs, to make them more applicable for students (Akbari-Kamrani, 2011; Mathur et al., 2004; Perrin & DeJoy, 2003; Pradhan& Strachan, 2003).

In Malaysia, while there is no legal compulsion for the school system to include sexual and reproductive health education (SRHE), the Ministry of Education faces several challenges in achieving the desirable outcome and objectives of SRHE. It is obvious that the success of the SRHE program that is part of the school curriculum does not live up to its expectations, as evidenced by the increasing incidences of social ills and misbehaviors involving youths in recent times (Low, 2009; Lee et al., 2006). Low (2009) found that adolescents in secondary schools did not appear to be getting the information they needed and most teachers did not want to deal with sensitive topics.

Teachers are the main presenters of education in schools. Clearly, the teacher’s knowledge, confidence and attitude are key factors in an advantageous sex education syllabus (Mathur et al., 2004). Currently, the situation in Malaysia does not only call for the need to equip teachers with the necessary knowledge and skills per se, but includes tackling issues pertaining to the quality of teaching and learning Science. Arguably, Malaysia, like many other countries in the world is confronted with the problem of inadequate trained science teachers especially in the teaching of Physics, Biology, Chemistry and Mathematics. As such, teachers of various educational backgrounds teaching science subjects were common in most schools. As a result, teachers with various subject majors’ background are often required to teach science subjects which they are not trained for (Osman at al., 2006). So far, little attention has focused toward teachers as key individual to deliver SBSRHE. Research assessing the status of this subject is required so as to fill the knowledge gap (Akbari-Kamrani, 2011).

In order to properly educate Malaysian adolescents, it is important to understand the constraints of the current school-based sexual and reproductive health education in secondary schools from the teacher’s viewpoint.

## 2. Method

### 2.1 Study Setting

This study was conducted in the Klang-Valley, an area in Malaysia comprising of its capital city, Kuala Lumpur and the adjoining cities and towns in the state of Selangor. The location was selected because of these main reasons: a) it fits the criteria of being a metropolis where the young are more exposed to the modern culture; b) this region includes the three main ethnic groups of Malaysia; c) it consist only of urban areas, and experiences a high percentage of migration (Akbari-Kamrani, 2011).

The education system in Malaysia comprises of these stages-pre-school, primary education, secondary education, tertiary education, and postgraduate studies. The education system is attained either through government schools or private schools mandated by the Malaysian law and falls under the purview of the Ministry of Education. Secondary education comprises of 5 years, Form 1 to Form 5 catering to the education needs of children aged between 13 to 18 years. Private schools in Malaysia mostly represent one out of the three main ethnic groups (Malays, Chinese and Indians). Therefore, in order to avoid biases, only government schools were approached with the understanding that they represent all the three main ethnic groups according to their genuine distribution in the country.

Sex education is referred to as Family Health Education (FHE) in Malaysian schools. Elements of FHE have been taught in secondary schools through the Physical and Health Education, Science, Additional Science, Biology, Moral Education subjects since the implementation of the Integrated Secondary School Curriculum in 1989.

### 2.2 Data Collection and Analysis

The data was drawn from a larger generic and descriptive qualitative study on “Assessment of school-based sexual and reproductive health education in secondary schools in Klang-Valley, MALAYSIA” (Akbari-Kamrani, 2011). This paper, however focused and closely examined the perspective of Science teachers in assessment of the current SBSRHE. Sexual health is a culturally sensitive topic in Malaysia, and therefore in-depth studies have not been widely conducted. However the researcher is of the opinion that in order to develop an appropriate SBSRHE curriculum, detailed information and a better understanding of the curriculum context of the subject is crucial. Towards that objective, in-depth interviews were conducted with science teachers to obtain insightful individual perspectives toward SBSRHE.

An open-ended semi-structured interview guide was employed. The interview guide was developed by the researcher based on existing literature. The main questions in the in-depth interview guide reflected the logical flow of topics expected in this study supplemented by probing questions to ensure that the rich information in significant areas was not missed. The average duration of the interview was one to one and a half hours depending on the willingness of the interviewee to talk and the time constraints of the interviewee. All the interviews were tape –recorded with the consent of the interviewee. All of the ethics of privacy and confidentiality were listed to them and they were informed that the tape recording was for research purposes only, and that the tapes will not contain personal references. Except for one interview that was conducted in the teacher’s office, all of the interviews were conducted in the school counseling room. The interviews were scheduled at the teacher convenience and minor adjustments were allowed due to unforeseen circumstances.

Preliminary data collection was carried out for two main reasons, firstly to familiarize the researcher with the process of conducting interviews and more importantly to pilot the research questions. Subsequently, the interview guide was refined and retested to ensure clarity and reliability of the questions.

Purposive sampling was used to select participants for conducting interviews. In purposive sampling the goal is to select cases that are likely to be ‘information-rich’ with respect to the purposes of the study (Gall et al., 2007, p. 218). In other words the science teachers were included in the study because they had a particular perspective and certain insights that would inform the research. The interviews for this study proceeded until the researcher noted that no new data was generated and that the teachers were expressing similar points in their interviews (Bogdan, & Biklen, 2007; Creswell, 2009).

Because the data collected for this study were in the form of a text, thematic analysis was employed. Thematic analysis is defined as the deduction of qualitative information and the effort to make sense of a great volume of qualitative material as well as attempts to identify core consistencies and meanings (Patton, 2002).

Teacher interview data were collected first. Broad themes and subcategories emerged from the interview transcript. In relation to categorizing data, Merriam (2001) discusses the challenge of constructing categories or themes that capture some recurring patterns and that these categories “are most commonly constructed through the constant comparative method of data analysis” (p. 179). Merriam (2001) stated that “category construction is data analysis” (p. 180).

Merriam (2001) further states that category construction begins with reading the first interview transcript and jotting down notes, comments, observations, and questions in the margins. This information becomes then the most important aspect of the data. Next, Merriam (2001) suggests moving to the next set of data and scanning it in the same way and comparing this information to the previous set and continuing on in that manner until themes emerge.

### 2.3 Ethical Issues and Trustworthiness

This study obtained the approval from the Ministry of Education and the Ethics Committee of the Faculty of Medicine and Health Sciences, Universiti Putra Malaysia.

To ensure rigor, the principals of trustworthiness were applied. To enhance credibility of this study, the triangulation method was applied to data collection methods and data sources. In the triangulation, the findings should appear consistent across the different data collection methods (Patton, 2002). Also, transferability was enabled by providing thick descriptions to enable the reader to decide whether the findings can be applied to their situation.

## 3. Findings

### 3.1 Socio-Demographic Characteristics of Participants

Twenty eight science teachers between the ages of 28-49 years participated in this study. All except one was married with children and the number of children varied from two to five. All the teachers were female, reflecting the high ratio of female teachers in Malaysia. In terms of the experience it varied from novice teachers with 2 years to senior teacher of 21 years, resulting in an average of 10 years.

### 3.2 Identified Themes

The science teachers responded to the question (What is the perspective of teachers regarding the constraints of the current school-based sexual and reproductive health education in secondary schools of Klang-Valley Malaysia?). The most prominent emerging themes were: “Teacher Weakness”, “School Constraints” and” Parental Constraints”.

#### 3.2.1 Teacher weakness

One of the major barriers identified were the teachers themselves. The teaching strategy and capacity of the teachers were two important factors in the effective delivery of sexual and reproductive health education.

3.2.1.1 Inappropriate Teaching Strategy

Typically, most teachers in this study used the classic approach in teaching sexual and reproductive health, in which the students were made to memorize facts and sit for exams. This would imply that these teachers were rather conservative regarding the subject of sex and sexual health:

*On the other side, students more try to memorize and sit for the exam. Teachers only explain about hormones and pregnancy and about another item not speak openly. But they must know to learn from their life (T17-teacher)*.

*Although there is this syllabus, but classic teaching is not very effective. A lack of interest and participation amongst students is one reason for not changing this strategy (T24-teacher)*.

3.2.1.2 Inadequate Teacher Capacity

Considering the sensitivity of the subject, the ability of the teacher to control the class is very important. Inexperienced teacher and teacher’s gender have been recognized as two important obstacles.

3.2.1.2.1 Lack of Experience

Evidence from the study shows that a more experienced teacher would be able to control a class better as compared to a younger teacher with less experience in teaching. Being unmarried may also be a disadvantage to a teacher teaching SBSRHE as single teachers may find talking about sexual matters rather difficult and embarrassing.

*I didn’t receive any training in university about school-based sexual and reproductive health education. In university only general subject there is that it was not enough. Certainly, my knowledge is not enough. Every day I hear about social problems, especially about adolescents that my knowledge is not enough for this. I need to be trained about this special topic and going deep. Now in Ministry of education there is not any training or workshop for school-based sexual and reproductive health education. I am young and not married (T14-teacher)*.

*Science teachers are not experienced. They are always young and not married. When they come to class, they have learned, but emotional control in mind. Many teachers shy, especially at mixed classes and especially about sensitive organs. Students may be laughing and be a jockey. Teachers cannot be open and must be very serious. Thus, they have to take easy this part because it’s jockey to them (T6-teacher)*.

3.2.1.2.2 Teacher’s Gender

The teacher’s gender is also an important factor, seeing how awkward and difficult it is for a female teacher to teach a class full of male students and vice versa. This is a very pertinent issue as the current ratio is -20:80-of male to female teachers in Malaysia and as demonstrated in the study above, all 18 Science teachers in this study were female.

*Teachers are young and sex or gender of them is very important. Equal sex is good. Teachers in K.L are almost female, but in out of K.L are male. Yes, opposite sex of teacher is one barrier (T27-teacher)*.

#### 3.2.2 School Constraints

The lack of co-operation from the school administration is another barrier in delivering sexual and reproductive health education. Limited resources and mixed classes were referred to as barriers.

3.2.2.1 Limited Resources

Teachers are faced with limited resources in teaching sexual and reproductive health. The only available resources are text books and an animation, CD prepared by the Ministry of Education. Also, appropriate resources do not exist in school libraries for this topic. The lack of resource as well as clear guidelines on innovative pedagogy of teaching leaves many teachers rather unsure of how best to explain the topics in School-Based Sexual and Reproductive Health Education. All of these teachers are Science teachers, but received no proper training for the teaching of sexual and reproductive health education.

3.2.2.2 Mixed Classes

Mixed classes are also an obstacle because Malaysian students, especially girls, tend to be shy around their male counterparts and are reluctant to ask questions:

*When these classes are mixed they don’t ask any question and they are embarrassed. They want to ask, but they embarrassed each other. Always they wait everybody gone, then come to ask you (T12-teacher)*.

*I believe that when we speak in separate classes it is more effective we can speak more freely. The cooperation of students will increase and a better feeling occurs (T23-teacher)*.

It might be a worthwhile idea for schools to form single sex classes when teaching SBSRHE to create a more conducive learning environment for the different genders as well as the teacher.

#### 3.2.3 Parental Constraints

Although parents entrusted the school with the education of their children, they consider the subject of sex off-limits and expect teachers to be conservative on this matter:

*Parent-teacher association (PTA) complain about the teacher that they teach too much and out of the syllabus (T1-teacher)*.

*One problem there is. Parents afraid we tell truth about sex and sometimes complain about this (T10-teacher)*.

The lack of knowledge of SBSRHE among parents and Malaysian society is an issue that should be addressed in order to improve the delivery of this subject. Parents may be unaware of the objectives of School-Based Sexual and Reproductive Health Education and therefore there exist an element of mistrust and lack of cooperation.

## 4. Discussion

Regarding the constraints of the current school-based sexual and reproductive health education (SBSRHE) program, the majority of participants named the teaching strategy and capacity of teachers, the lack of co-operation from the school and parents, limited resources in teaching and students as some of the challenges. This concern is supported by the results of other studies which found that the barriers to effective skills-based health education included inadequate teacher training, insufficient targeting of specific skills, lack of quality teaching materials and participatory methods, inconsistent messages, low intensity and scale, and poor monitoring and evaluation (Nitirat, 2007; Plummer et al., 2007; Kantabutra & Tang, 2006).

This study also highlighted that the traditional approach in delivering SBSRHE is another barrier. This finding is consistent with previous research which revealed that conventional didactic lectures are not effective in teaching adolescents and have limited use in sex education (Measor, 2004). In contrast with traditional didactic teaching methods, active, informal, personalized, and participatory learning methods that are culturally appropriate have been proven to be most effective in influencing the development of attitudes and changing sexual behavior. The findings from this study clearly demonstrated that the lack of interest and participation amongst students is one key reason to change this strategy. One important insight into the key elements that lead to greater behavioral changes amongst adolescents is the use of interactive methods, skill-based activities, and real-life situations (Tijuana et al., 2002). As stated in Bandura’s model (1986), adolescents are products and producers of SRH education in school as they select whom they interact with and how much they participate in activities. This influences the way they experience their environment and effects formation and initiation of behaviors. For example, there is a need to develop a less formal and restrained contact with students, as this could increase the program’s effectiveness. Active in teaching approaches such as small group discussions, updated videos and discussions, anonymous question sessions, drama and role-play are highly valued by students, thus emphasizing the importance of a more up-to-date interactive teaching strategy (Allen, 2005; Mathur et al., 2004; Pokharel et al., 2006).

Participating teachers also reported that they did not want to deal with sensitive topics and feared censure by their colleagues. Similarly, Bragg (2006), Cohen et al. (2004), and Pokharel et al. (2006) reported that teachers fear that too much sex education is inappropriate or too explicit and consequently, they may be reprimanded by parents or the school management. Usually, cultural norms dictate the types of acceptable topics to be taught and also who may teach them.

The present study revealed an important shortcoming in the delivery of SBSRHE in Malaysia, which is the non-existence of in-service training for teachers assigned to teach this subject. The Ministry of Education and certain non-government organizations (e.g. Malaysian AIDS Council) provides some short in-service courses focusing on the biological and scientific aspects of sexual and reproductive health. However, skills-based approaches are becoming increasingly common. Curriculum based programs are essential, but it is also important to correctly train instructors to tackle these sensitive issues in order to gain adolescent confidence (Suneth et al., 2008). Teachers need to be trained and equipped with the skills to deliver sex education in an age appropriate, gender sensitive and youth-friendly manner (Senderowit, 2000).

The teacher interviews showed that mixed classes were a key obstacle because Malaysian students, especially girls, found it hard to openly express themselves in the presence of their male classmates. A previous study by Buston et al. (2002) also found that some girls felt too embarrassed to talk in front of boys. Boys, on the other hand expressed concern and fear of being ridiculed by girls if they participated too much (Strange et al., 2003). Most students in a mixed-gender class preferred a single-gender class to reduce embarrassment. Surveys of students also found that the majority of girls and far fewer boys prefer single gender classes (Allen, 2005; Strange et al., 2003). In the studies by Cohen et al. (2004) and Kehily (2002), there was evidence that teachers struggled with varying ranges of student maturity and knowledge, lack of interest and shyness.

According to Bandura (1986), personal factors such as age or gender may influence levels of comfort, attentiveness, and participation during school-based sexual and reproductive health classes. The mixed-sex context is particularly problematic. Girls’ and boys’ different responses to the lessons compounds the discomfort felt about how members of the opposite sex will react to one’s contributions (Buston et al., 2001). In an environment where macho performances (of both students and teachers) reflect the wish of girls (and boys) to be desired, teaching and talking about sexuality becomes difficult and even unsafe (Epstein, & Johnson, 1998). It is important that teachers understand the pedagogy of SRH education as a critical and transformative practice.

## 5. Conclusion

It can be concluded that the quality of teaching sex and reproductive health education in Malaysia is unsatisfactory due to inadequate preparation of teachers for such instruction, lack of adequate teaching materials and lack of school and community support. In this respect, sexual health education in schools is a specialized aspect of health promotion requiring detailed attention from its planning through to the delivery. Effective teacher training is required for successful sexual health promotion in classrooms and this need to be acknowledged in the administrative budget planning. Educational activities should target teachers as well, which would enable them to play a beneficial role in the sexual and reproductive health of the younger generation.
